# Maternal Pregestational Diabetes Contributes to Neural Tube Defects in Mouse Fetuses Through H4K5ac-Mediated Regulation of Focal Adhesion Pathway

**DOI:** 10.3390/genes17060671

**Published:** 2026-06-08

**Authors:** Jiaxin Cheng, Kexin Zhang, Shuangshuang Yang, Baoling Bai, Qin Zhang

**Affiliations:** 1 Key Laboratory of Child Development and Nutriomics, Capital Institute of Pediatrics, Chinese Academy of Medical Sciences & Peking Union Medical College, Beijing 100020, China; 2 Key Laboratory of Child Development and Nutriomics, Capital Center for Children’s Health, Capital Medical University, Capital Institute of Pediatrics, Beijing 100020, Chinabaoxiang8802@126.com (B.B.)

**Keywords:** maternal diabetes, neural tube defects, mass spectrometry, ChIP-seq, H4K5 acetylation

## Abstract

**Objectives**: To investigate the potential mechanisms of maternal pregestational diabetes-induced neural tube defects (NTDs) by integrating proteomic data and histone H4 lysine 5 acetylation (H4K5ac) ChIP-seq data from the mouse model. **Methods**: The diabetic mouse model was established by intraperitoneal injection of streptozotocin (STZ) into female friend leukemia virus B strain (FVB) mice, with subsequent blood glucose monitoring. Diabetic females were then mated with healthy males, and embryonic tissues were collected on embryonic day 9.5. Among the embryos obtained from diabetic pregnancies, six NTDs embryos and six control embryos were selected for protein expression profiling using tandem mass tag (TMT)-labeled liquid chromatography-tandem mass spectrometry (LC-MS/MS), as well as for assessment of H4K5ac modification by ChIP-seq. Multi-omics integration was performed to identify common differentially expressed genes, followed by functional enrichment analysis. Key genes were validated using RT-qPCR. **Results**: Proteomic analysis revealed that differentially expressed proteins were significantly enriched in focal adhesion pathway. Protein–protein interaction (PPI) network analysis indicated that these proteins (e.g., Integrin alpha 3 (Itga3), glycogen synthase kinase 3 beta (Gsk3b), mitogen-activated protein kinase 9 (Mapk9)) were associated with focal adhesion and cytoskeletal functions. Integrated multi-omics analysis identified 923 common differentially expressed genes, which were also significantly enriched in focal adhesion pathway. Within this pathway, the protein expression levels of Itga3, Gsk3b, and Mapk9 exhibited a consistent co-variation trend with H4K5ac enrichment. RT-qPCR results confirmed that *Itga3* was significantly up-regulated, while *Gsk3b* was down-regulated in the NTDs group (*p* < 0.05). **Conclusions**: Maternal pregestational diabetes may contribute to NTDs by disrupting cytoskeletal reorganization, cell adhesion, and migration processes. This disruption is likely mediated through H4K5ac-regulated expression of key focal adhesion pathway genes such as *Itga3* and *Gsk3b*.

## 1. Introduction

Neural tube defects (NTDs) are the most common congenital malformations of the central nervous system [1,2], and pregestational diabetes mellitus (PGDM) is a significant risk factor [3,4]. The incidence of malformations in offspring of mothers with PGDM is 4–10 times higher than in non-diabetic pregnancies [5], with spina bifida and anencephaly being the most common (OR = 2.34) [6,7]. Clinical studies have confirmed that strict glycemic control during the first trimester reduces the fetal malformation rate from 10.9% to 1.2% [8]. Therefore, it is of great importance to elucidate the molecular mechanisms underlying maternal PGDM-induced NTDs.

Previous proteomic studies have revealed key molecular events in neural tube development. In curly tail(ct/ct) homozygous mice, abnormal migration of Laminin B1 is observed at E10.5, suggesting that it may be a potential genetic modifier of NTDs [9]. In the all-trans retinoic acid (ATRA)-induced rat model of spina bifida, matrix-assisted laser desorption/ionization time-of-flight mass spectrometry (MALDI-TOF MS) identified aberrant expression of Collapsin response mediator protein 4 (Crmp4), Heat shock protein 70 (Hsp70), and Calponin-3, which are involved in signal transduction, transcriptional regulation, and protein folding, and may collectively contribute to spina bifida [10]. Moreover, liquid chromatography-mass spectrometry (LC/MS) studies have shown that Coronin-1A and Dynamin-2 are significantly downregulated in exosomes from spina bifida samples, indicating their potential as early biomarkers for NTDs screening and diagnosis [11]. Immunoprecipitation-mass spectrometry (IP-MS) results further confirmed that Sarcolemmal membrane-associated protein 3 (SLMAP3) interacts with cytoskeletal components such as actin, microtubules, and intermediate filaments, suggesting that the cytoskeleton is a primary target of SLMAP3 during embryonic brain development [12]. However, the proteomic changes underlying maternal PGDM-induced NTDs remain unclear.

Diabetes-induced embryonic malformations involve multiple mechanisms [13], among which aberrant epigenetic modifications are closely associated with PGDM-induced NTDs [14,15]. Studies have shown that histone acetylation modifications are critical for neural tube closure [16,17]. Yu et al. found that suppression of Sirtuin (SIRT) expression and elevated histone H3 acetylation levels under high-glucose exposure are important mechanisms leading to NTDs [14]. In addition, the enrichment level of histone H4 lysine 5 acetylation(H4K5ac) was significantly altered in the embryonic genome of diabetic or high-fat diet-fed female mice [18]. Our previous studies further showed increased H4K5ac modification in human NTDs embryonic brain tissues. Moreover, using mouse models and cell experiments, we confirmed that both maternal diabetes and in vitro high-glucose exposure significantly elevate H4K5ac levels [19,20]. This suggests that H4K5ac may be an important epigenetic factor in maternal PGDM-induced NTDs. However, it remains unclear how maternal diabetes affects neural tube closure through H4K5ac modification.

Therefore, this study aimed to integrate ChIP-seq and proteomics to dissect both the involvement of embryonic protein expression in maternal diabetes-induced NTDs and the regulatory function of H4K5ac modification on key genes.

## 2. Materials and Methods

### 2.1. Animal Experiments

This study was approved by the Medical Ethics Committee of the Capital Institute of Pediatrics, Beijing, China (Approval No. DWLL2014007). It was conducted at the same institute from September 2017 to December 2018.

A priori sample size estimation was based on a preliminary study showing an NTDs incidence of approximately 14% [20,21,22]. Given the average litter size of friend leukemia virus B strain (FVB) mice (8–12 embryos per dam) and the need for at least six NTDs-affected embryos for multi-omics analyses, we calculated that at least 43 total embryos (≈4–5 pregnant dams) were required. To account for potential infertility and fetal loss, we ultimately enrolled 40 female mice per group, ensuring a high probability of achieving the target sample size and supporting the feasibility of the study objectives.

A total of 40 female FVB mice aged 7–9 weeks were used in this study. Diabetes was induced by two intraperitoneal injections of streptozotocin (STZ, CAS 18883-66-4, Sigma-Aldrich, St. Louis, MO, USA) at an interval of one week. STZ was dissolved in 100 mM citrate buffer and administered at a dose of 100 mg/kg body weight. Control mice (*n* = 40) received an equal volume of citrate buffer alone. Following a 12 h fast after STZ injection, mice with a sustained blood glucose concentration exceeding 14 mM (250 mg/dL) were considered diabetic. In total, 28 mice were successfully modeled. Diabetic female mice were mated with male FVB mice aged 8–11 weeks at a female: male ratio of 3:1. The day of vaginal plug detection was designated as embryonic day 0.5 (E0.5). On embryonic day 9.5 (E9.5), pregnant mice were euthanized by CO_2_ inhalation, and embryonic samples were collected for subsequent analyses. A total of 23 female mice became pregnant and produced 149 embryos, of which 20 exhibited NTDs, corresponding to a malformation rate of 13.4% (20/149). From these 20 NTDs-affected embryos, six were randomly selected for subsequent validation experiments.

To minimize potential bias in the study, the following measures were implemented. First, female FVB mice were randomly assigned to the diabetes induction group or the control group using a computer-generated random number sequence. Second, all procedures, including animal husbandry, STZ injections, blood glucose measurements, and tissue collections, were carried out according to strictly standardized protocols to reduce technical variation. Third, no animals or data points were excluded from the analysis except for those that failed to meet the pre-defined diabetes criterion (blood glucose > 14 mM). Such exclusions were applied equally to both groups and are explicitly reported in the results.

### 2.2. Liquid Chromatography-Tandem Mass Spectrometry (LC-MS/MS)

#### 2.2.1. Total Protein Extraction from Tissues

Total proteins were extracted from mouse E9.5 embryonic brain tissues as previously described [23], using the One Step Animal Tissue Active Protein Extraction Kit (C500006; Sangon Biotech, Shanghai, China). Briefly, 0.5 μL protease inhibitor, 2.5 μL phosphatase inhibitor, 0.5 μL dithiothreitol, and 5 μL phenylmethylsulfonyl fluoride were sequentially added to 500 μL pre-cooled lysis buffer. Six independent brain tissue samples were taken from each of the control group and the NTDs group, and each sample was weighed to 100 mg. The tissues were cut into small pieces and washed twice with 1× phosphate-buffered saline. After centrifugation, 500 μL of pre-cooled lysis buffer was added, and the samples were homogenized 15–30 times. The samples were then sonicated 4–6 times for 30 s each with a 1 min interval between pulses. Subsequently, the samples were centrifuged at 15,000× *g* for 10 min at 4 °C, and the supernatants were transferred to new pre-cooled centrifuge tubes. Protein concentrations were determined using the BCA assay kit (P0012; Beyotime, Shanghai, China). The six brain tissue samples from each group were pooled in groups of three to generate one mixed sample, resulting in C-1 and C-2 (from the control group) as well as NTD-1 and NTD-2 (from the NTDs group). The total protein amount of each pooled sample was adjusted to 100 μg. Finally, the four pooled samples (C-1, C-2, NTD-1, NTD-2) were obtained and used for subsequent protein digestion experiments.

#### 2.2.2. Protein Digestion and Tandem Mass Tag (TMT) Labeling

Four pooled samples (100 μg each) were each mixed with 6 μL of trypsin (Promega, Madison, WI, USA) and 100 μL of 50 mM triethylammonium bicarbonate buffer, and then incubated at 37 °C for at least 20 h. After digestion, protein concentrations were measured again using the BCA assay kit (P0012; Beyotime, Shanghai, China). Subsequently, 50 μg of each sample was labeled with a distinct TMT label, as follows: C-1 was labeled with TMT126, C-2 with TMT127, NTD-1 with TMT128, and NTD-2 with TMT129 (10-plex™ Isobaric Mass Tag Labeling Kit, 90113CH; Thermo Fisher Scientific, Waltham, MA, USA). 20 μg of each labeled sample were pooled, and the pooled labeled peptides were fractionated using the fractionation column for MS analysis.

#### 2.2.3. Nano-High-Performance Liquid Chromatography/Mass Spectrometry (Nano-HPLC/MS) Analysis

The fractionated samples were diluted with buffer A (98% ultrapure water, 5% acetonitrile, and 0.05% formic acid) and then loaded onto an UltiMate 3000 RSLC nano system (Dionex, Sunnyvale, CA, USA). Samples were analyzed using the QExactive HF Orbitrap mass spectrometer (Thermo Fisher Scientific, Waltham, MA, USA). The ion spray voltage was maintained at 2.2 kV, the capillary temperature at 320 °C, and the S-Lens RF level at 60. In data-dependent acquisition mode, MS scans (*m*/*z* 300–1500) were acquired at a resolution of 60,000, and the 20 most intense precursor ions were isolated. The TMT-labeled peptides were fragmented by higher-energy collisional dissociation, and MS/MS spectra were acquired at a resolution of 30,000.

#### 2.2.4. Protein Identification and Quantification

Data analysis was performed using Proteome Discoverer software (version 2.1.0.81; Thermo Fisher Scientific, Waltham, MA, USA). The human whole-protein database was downloaded from the UniProt website (https://www.uniprot.org/) for searching. Search parameters were as follows: trypsin digestion with a maximum of two missed cleavages; precursor mass tolerance of 10 ppm; fragment mass tolerance of 0.02 Da; relative peak intensity calculated based on signal-to-noise ratio; and normalization of each TMT label based on total peptide amount. Data were considered reliable when *p* < 0.0017 and the false discovery rate (FDR) < 0.01. After database retrieval, the data were exported in Excel format (version 2016; Microsoft, Redmond, WA, USA) and annotated according to the group names (C-1, C-2, NTD-1, NTD-2) for subsequent differential analysis and graph generation.

### 2.3. Chromatin Immunoprecipitation Followed by Sequencing (ChIP-seq)

ChIP experiments were performed using the SimpleChIP^®^ Enzymatic Chromatin IP Kit (Cell Signaling Technology, Danvers, MA, USA) according to the manufacturer’s instructions, as described previously [24]. Formaldehyde-crosslinked chromatin was obtained from fetal mouse brain tissues. Crosslinked chromatin was immunoprecipitated overnight at 4 °C with an antibody against H4K5ac (Cell Signaling Technology, Danvers, MA, USA). Normal rabbit IgG (Cell Signaling Technology, Danvers, MA, USA) was used as a negative control. The immunoprecipitated DNA was subjected to sequencing. Deep whole-genome DNA sequencing was performed by BGI (Shenzhen, China; https://www.genomics.cn/). Raw sequencing image data were processed using the Illumina analysis pipeline (version 1.0; Illumina, San Diego, CA, USA). Sequencing reads were aligned to the mouse (*Mus musculus*) reference genome (UCSC, mm9 version) using Bowtie 2 software (version 2.4.0), and then subjected to peak calling with MACS (Model-based Analysis of ChIP-Seq; version 2.2.7.1, https://github.com/taoliu/MACS (accessed on 1 July 2019)). Enriched binding peaks were identified by comparison with control input samples.

### 2.4. Reverse Transcription-Quantitative Polymerase Chain Reaction (RT-qPCR)

Total RNA was extracted using TRIzol reagent (Invitrogen, Waltham, MA, USA) and reverse-transcribed into cDNA using the Transcript First-Strand cDNA Synthesis Super Mix (TransGen Biotech, Beijing, China) according to the manufacturer’s recommendations. RT-qPCR was performed using Ultra-SYBR Mixture (abm, Richmond, BC, Canada). Relative mRNA levels in different groups were quantified using the QuantStudio™ 6 Flex RT-qPCR system(Thermo Fisher Scientific, Waltham, MA, USA). The reaction mixture contained forward and reverse primers (0.5 μL each), Ultra-SYBR Mixture (6 μL), cDNA (1 μL), and deionized water (3 μL). Gene expression was normalized to *β-actin*. The relative levels of mRNA transcripts were calculated using the classical ΔΔCt method. The primer sequences used in this study are shown in Table 1.

### 2.5. Bioinformatics and Statistical Analysis

The following criteria were used to screen for differentially expressed data. For ChIP-seq analysis, a fold change (FC) > 2 or < 0.5 with *p* < 0.045 was set as the threshold for H4K5ac-modified differentially bound genes, where FC > 2 indicated upregulated binding and FC < 0.5 indicated downregulated binding. For proteomics data, differentially expressed proteins (DEPs) were defined as those with FC > 1.3 or FC < 0.76 and *p* < 0.05, where FC > 1.3 represented upregulated DEPs and FC < 0.76 represented downregulated DEPs.

KEGG pathway enrichment and Gene Ontology (GO) annotation analyses were performed using the BioDeep platform (https://www.bioinformatics.com.cn/). WIKI pathway analysis of DEPs was conducted using the DAVID database. KEGG and WIKI analyses identify the metabolic and signal transduction pathways in which the differentially expressed molecules are involved. GO analysis systematically annotates their functions from three perspectives: biological process, cellular component, and molecular function, thereby clarifying the specific biological processes they participate in.

Statistical analyses were performed using GraphPad Prism 9.0 and SPSS 22.0 software. For comparisons among multiple groups, one-way ANOVA was used for data with a normal distribution, and the Kruskal–Wallis test was used for non-normally distributed data. Comparisons between two groups were performed using Student’s *t*-test. A *p* value < 0.05 was considered statistically significant.

## 3. Results

### 3.1. Dysregulation of Focal Adhesion and Cytoskeleton Regulatory Protein Expression in the Brain of E9.5 NTDs Fetuses Induced by Pregestational Diabetic Dams

**Figure 1:**
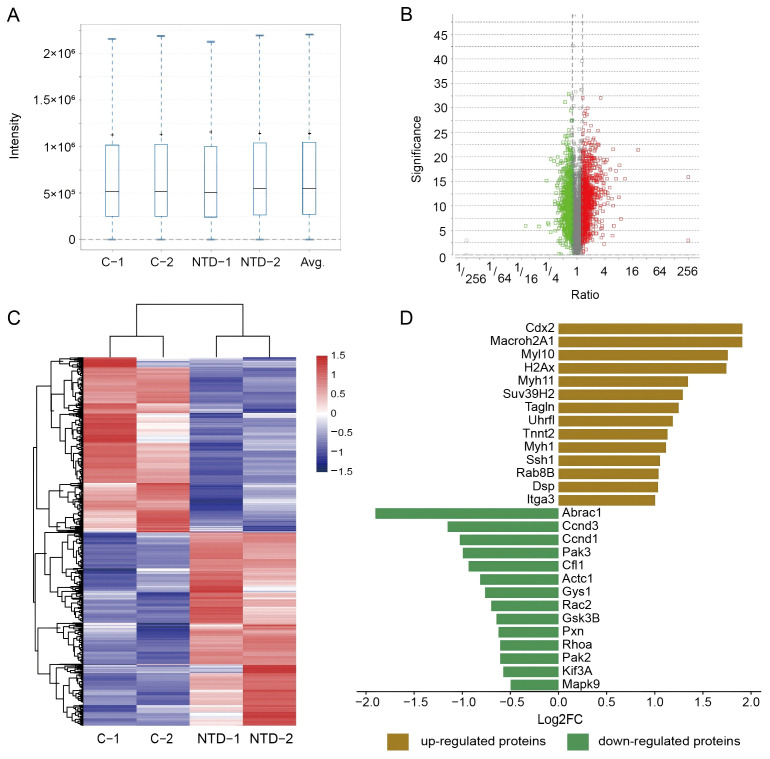
Protein expression profiles in brain tissues of E9.5 NTDs embryos from pregestational diabetic dams. (**A**) Protein abundance between the control (*n* = 6) and NTDs (*n* = 6) groups. (**B**) Volcano plot of differentially expressed proteins (DEPs). Green squares represent proteins with fold change (FC) > 1.3 and *p* < 0.05; red squares represent proteins with FC < 0.76 and *p* < 0.05; gray squares represent proteins that did not meet these thresholds. (**C**) Hierarchical clustering heatmap of protein expression patterns in the two groups. (**D**) The number of up- and down-regulated DEPs in the NTDs group compared to the control group.

Total proteins were extracted from mouse E9.5 embryonic brain tissue samples of the control group and the NTDs group induced by pregestational diabetic dams (six biological replicates per group), followed by TMT-labeled proteomic analysis. A total of 2797 proteins were identified. Figure 1A showed the consistency of labeling results across groups. Figure 1B presented the volcano plot of DEPs, defined by FC > 1.3 or < 0.76 (*p* < 0.05). In total, 1868 DEPs were identified, of which 1016 were significantly upregulated and 852 were significantly downregulated. Furthermore, clustering analysis revealed a distinct separation in protein expression patterns between the two groups (Figure 1C).

Figure 1D listed the DEPs in the NTDs group compared with the control group. Upregulated proteins included Ssh1 and Tagln (actin dynamics regulators), Itga3 (mediating ECM-cytoskeleton signal transduction), Rab8b (involved in vesicle transport and cell polarity establishment), H2ax (DNA damage repair protein), Dsp (cell–cell adhesion junction protein), as well as Myl10, Myh10, and Myh11 (core components of the cytoskeleton and contractile units). Downregulated proteins included cyclins Ccnd1/Ccnd3, Gsk3b (a key regulator of the Wnt/β-catenin pathway), Klf3a (ciliary transport-related protein), Cfl1, Pak2 and Pak3 (regulating cytoskeletal reorganization), Pxn (focal adhesion scaffold protein), Mapk9 (involved in cellular stress response), and Gys1 (glycogen metabolic enzyme).

**Figure 2:**
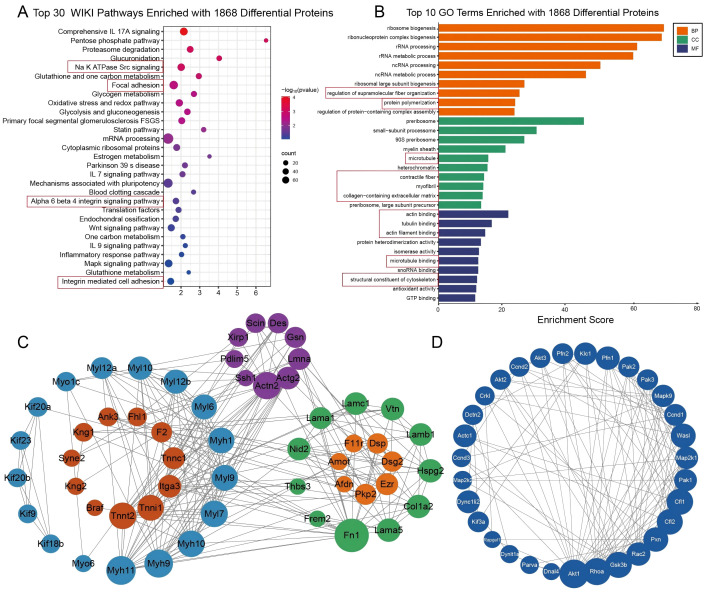
Functional analysis of differentially expressed proteins (DEPs) between the control and NTDs groups. (**A**) WIKI pathway enrichment analysis of DEPs. (**B**) Gene Ontology (GO) functional enrichment analysis of DEPs. Red boxes in (**A**,**B**) highlight pathways of particular interest, which are closely related to focal adhesion and cytoskeletal regulation. (**C**,**D**) Protein–protein interaction (PPI) networks of up-regulated (**C**) and down-regulated (**D**) DEPs.

To further characterize the functions of the DEPs, we performed WIKI pathway enrichment and GO annotation analyses on these 1868 DEPs (Figure 2A,B). The WIKI analysis revealed significant enrichment of pathways including focal adhesion, integrin-mediated cell adhesion, the α6β4 integrin signaling pathway, and the Na+/K+-ATPase-Src signaling pathway (Figure 2A). Focal adhesions are core structures that connect cells to the extracellular matrix (ECM), anchoring the intracellular actin cytoskeleton to the ECM. Integrins, as central components of focal adhesions, recruit and activate downstream kinases such as Focal adhesion kinase (FAK) and Proto-oncogene tyrosine-protein kinase src (Src) to directly regulate cytoskeletal reorganization (e.g., actin polymerization, microfilament crosslinking, and contraction), thereby coordinating cell adhesion, migration, and morphological remodeling [25,26]. This suggests that disruption of focal adhesion and related signaling pathways may contribute to the pathogenesis of NTDs by impairing cytoskeletal dynamics. GO analysis provided supporting evidence from another perspective: these proteins were mainly involved in cytoskeletal assembly and the subsequent cell adhesion and migration processes (Figure 2B). Together, these results indicate that focal adhesion dysfunction and the resulting cytoskeletal abnormalities may play a critical role in maternal diabetes-induced NTDs.

To explore the potential role of focal adhesion-mediated cytoskeletal regulation in NTDs, we constructed protein–protein interaction (PPI) networks. Upregulated DEPs were found to regulate cytoskeletal reorganization and related cellular behaviors via focal adhesion-mediated signal transduction. These proteins could be classified into five functionally synergistic core modules: ECM components, cell adhesion junction proteins, signal transduction and regulatory proteins, cytoskeletal structural and regulatory proteins, and molecular motors and motor proteins. Notably, several known NTDs-associated hub molecules (e.g., Itga3, Hspg2, Lama5, Lamc1, and Frem2) were located within these modules (Figure 2C). Meanwhile, downregulated proteins were also significantly enriched in signal transduction and cytoskeleton-related pathways, and included multiple focal adhesion-associated proteins such as Gsk3b, Mapk9, and Ccnd1/2/3 (Figure 2D). These results suggest that dysregulated expression of key proteins in the focal adhesion pathway may collectively lead to NTDs by disrupting cytoskeletal dynamics.

### 3.2. H4K5ac Regulates the Focal Adhesion Pathway Genes Itga3 and Gsk3b, Thereby Contributing to the Pathogenesis of Maternal Pregestational Diabetes-Induced NTDs

**Figure 3:**
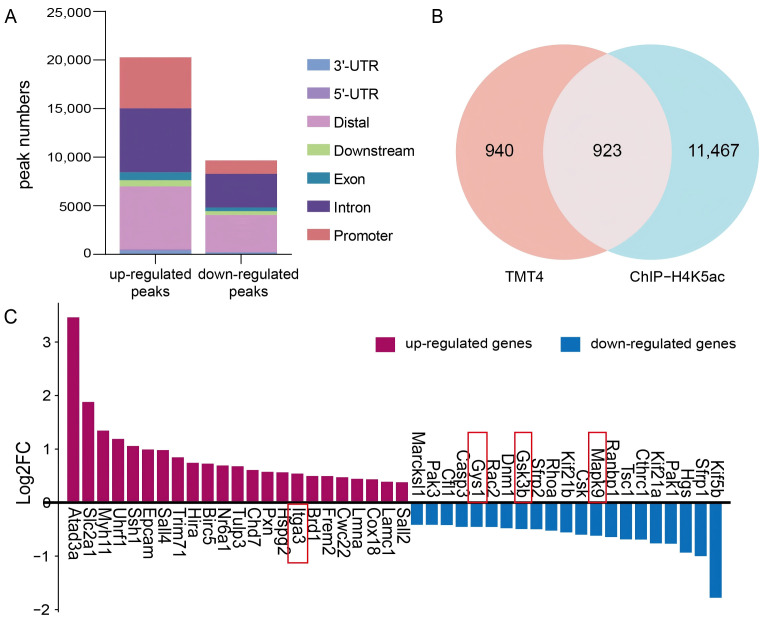
Integrated analysis of proteomic and ChIP-seq data. (**A**) Distribution of H4K5ac differential peaks across genomic functional regions. (**B**) Venn diagram showing the overlap of differentially expressed genes (DEGs) between ChIP-seq and proteomic analyses. (**C**) Up- and down-regulated genes associated with NTDs, screened from the common DEGs. Red boxes indicate focal adhesion-related genes of particular interest.

H4K5ac is a typical transcription-activating modification that is often enriched at gene promoters and coding regions to promote gene expression [27,28]. Our previous studies have suggested that histone H4K5ac may play a key role in maternal diabetes-induced NTDs. To determine whether H4K5ac regulates genes associated with NTDs, we performed ChIP-seq to profile genome-wide H4K5ac enrichment in mouse embryonic brain tissues. Compared with the control group, the NTDs group exhibited 202,086 downregulated peaks and 9683 upregulated peaks. These differentially enriched peaks were mainly distributed in promoter regions, distal intergenic regions, and gene downstream regions (Figure 3A).

To further investigate their functions, we integrated the proteomics data with the H4K5ac ChIP-seq data and identified 923 differentially expressed genes (DEGs) that were common to both datasets, forming a core gene set potentially regulated by H4K5ac (Figure 3B). Within this set, we identified several known NTDs-associated genes (including *Itga3, Gys1, Gsk3b, Mapk9,* all of which are involved in the focal adhesion pathway), and their protein expression levels showed a consistent trend with H4K5ac enrichment [29] (Figure 3C, Table 2).

**Figure 4:**
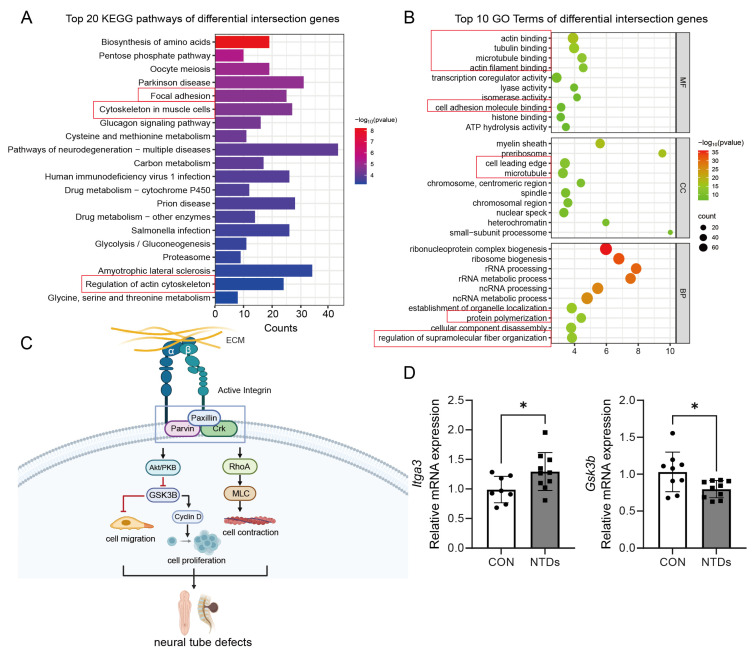
Functional analysis and experimental validation of common differentially expressed genes (DEGs). (**A**) KEGG pathway enrichment analysis of DEGs. (**B**) Gene Ontology (GO) functional enrichment analysis of DEGs. Red boxes in (**A**,**B**) highlight pathways of particular interest, which are closely related to focal adhesion and cytoskeletal regulation. (**C**) Diagram of the possible mechanism underlying NTDs in this study. (**D**) RT-qPCR validation of the relative mRNA expression of *Itga3* and *Gsk3b* genes from focal adhesion pathway. (Data are from three independent biological experiments. The scatter points represent the technical measurements within each biological replicate. * *p* < 0.05).

We performed KEGG pathway and GO enrichment analyses on this gene set. The top 20 KEGG pathways showed that these genes were significantly enriched in pathways such as focal adhesion, regulation of actin cytoskeleton, as well as dynein, ECM-receptor interaction, tight junction, and adherens junction (Figure 4A). GO analysis revealed their functional characteristics from multiple dimensions: in the “cellular component” category, they were significantly enriched in structures such as the leading edge of the cell and microtubules; in the “molecular function” category, they mainly included tubulin binding, microtubule binding, cell adhesion molecule binding, actin binding and filamentous actin binding; in the “biological process” category, they were also significantly involved in processes such as protein polymerization (Figure 4B). Together, these results suggest that H4K5ac, as a key epigenetic regulator of NTDs, may regulate neural tube closure by mediating the expression of focal adhesion and cytoskeleton-related genes.

Given the regulatory effect of H4K5ac on focal adhesion pathway genes such as *Itga3* and *Gsk3b*, we performed RT-qPCR validation. The results showed that, compared with the control group, *Itga3* expression was significantly upregulated in the NTDs group, while *Gsk3b* expression was significantly downregulated (Figure 4C,D).

In summary, the hyperglycemic environment may regulate the expression of key focal adhesion pathway genes *Itga3* and *Gsk3b* via H4K5ac, thereby disrupting cytoskeletal reorganization, cell adhesion, and migration, and ultimately contributing to the pathogenesis of NTDs.

## 4. Discussion

In this study, we established the mouse model of NTDs induced by maternal pregestational diabetes and, by integrating proteomic and H4K5ac ChIP-seq data, linked NTDs pathogenesis to the focal adhesion pathway. Furthermore, we found that the expression of key genes in this pathway, *Itga3* and *Gsk3b*, is regulated by H4K5ac modification. These results suggest that maternal diabetes may disrupt cell adhesion dynamics and behavior through this epigenetic mechanism, thereby contributing to the development of NTDs.

Numerous studies have confirmed that dysfunction of focal adhesion and cytoskeletal regulatory proteins plays an important role in the pathogenesis of NTDs [30,31]. Consistent with this, our WIKI pathway enrichment, GO annotation, and PPI network analyses collectively indicate that high glucose exposure may lead to dysregulation of focal adhesion-related proteins (e.g., Itga3, Lamc1, Gsk3b, Mapk9, Ccnd3), disrupt focal adhesion signaling and the cytoskeletal dynamics regulated by this pathway, and thereby participate in the development of NTDs.

Focal adhesions are the primary anchoring sites between cells and the ECM. They not only provide mechanical linkages but also couple integrin-mediated signaling with actin cytoskeletal dynamics, thereby precisely regulating cell migration and collective cell behavior [32,33]. Studies have shown that loss of the focal adhesion protein Talin-1 disrupts cytoskeletal rearrangement and cell morphology, subsequently affecting vascular remodeling [34]. miR-27 has been demonstrated to promote pharyngeal arch cartilage development by downregulating FAK expression [35]. Moreover, regulators of the cytoskeleton, such as Rho GTPase-activating factors (e.g., βPix, DLC-1), participate in embryonic development by modulating focal adhesion dynamics and cell migration; their deficiency can lead to NTDs [36,37]. Previous studies have fully established the critical role of focal adhesion and cytoskeleton-related pathways in embryonic development. Building on this foundation, the present study reveals that maternal diabetes-induced NTDs are closely associated with the focal adhesion pathway, and that this association may be mediated by epigenetic regulation of the core pathway genes *Itga3* and *Gsk3b* via H4K5 acetylation.

Integrins are a family of glycoprotein receptors located on the cell surface, typically functioning as heterodimers composed of different α and β subunits. They link the ECM to the intracellular actin cytoskeleton and play essential roles in maintaining cell morphology, regulating cell adhesion, proliferation, differentiation, and other physiological processes [38]. Among them, ITGA3 is critical for embryonic development, particularly in nervous system formation. Studies have shown that ITGA3 is involved not only in neural precursor cell differentiation and development [39], but also in mediating the termination of Reelin (RELN)-guided, radial glia-dependent neuronal migration [40]. In addition, p53 deficiency significantly increases the incidence of NTDs in mouse embryos and is accompanied by marked downregulation of the *Itga3* gene [41]. In our study, abnormally high expression of Itga3 may lead to excessive cell adhesion and impaired migration, thereby contributing to the pathogenesis of NTDs induced by maternal pregestational diabetes.

GSK3 is a conserved Ser/Thr protein kinase that includes two isoforms, GSK3A and GSK3B, both highly enriched in brain tissue and critical for neural development and synaptic plasticity. *Gsk3b* knockout mice exhibited pronounced structural and functional abnormalities in the brain [42]. Furthermore, inhibition of Gsk3b and inositol monophosphatase activity disrupts inositol biosynthesis and cellular homeostasis, leading to lithium-induced NTDs [43]. In our maternal diabetes-induced NTDs mouse model, we also observed significant downregulation of *Gsk3b*, which was regulated by H4K5ac, suggesting that Gsk3b may play a key role in NTDs induced by maternal diabetes.

Based on previous and current evidence, ITGA3 specifically binds to its ligand laminin, thereby transducing ECM signals into the cell. This process relies on downstream proteins such as FAK and Src, which subsequently activate the PI3K-AKT signaling pathway. Activated AKT inhibits GSK3B through phosphorylation, leading to dysregulation of downstream targets such as β-catenin and cyclin D family proteins, ultimately resulting in dysfunction of cell adhesion and migration [44,45].

By integrating proteomic and H4K5ac ChIP-seq data, this study identified a potential link between specific histone modifications and gene expression regulation in the mouse model of NTDs induced by maternal pregestational diabetes, thereby providing new insights into the epigenetic regulatory mechanisms during neural tube closure. These findings suggest that key focal adhesion pathway proteins (e.g., ITGA3, GSK3B) have potential as early biomarkers for diabetes-associated cellular dysfunction. Moreover, because H4K5ac is a reversible modification, its upstream acetyltransferases/deacetylases or downstream effector proteins (e.g., ITGA3) may serve as potential therapeutic targets for intervention in hyperglycemia-induced pathological processes.

Several limitations of this study should be acknowledged. First, this study only examined a single developmental time point (E9.5); the dynamic changes in H4K5ac modification and focal adhesion pathway activity during the entire neurulation process remain unknown. Second, the potential contribution of other histone modifications (e.g., H3K9ac, H3K27ac) or non-histone epigenetic mechanisms was not explored, and we cannot exclude their involvement in maternal diabetes-induced NTDs. Third, all experiments were performed using a single mouse strain (FVB), and the results may not be directly transferable to other genetic backgrounds. Fourth, this study is based primarily on trend-based correlations from omics data; direct statistical associations between TMT protein quantification and ChIP-seq signals require further validation in sample sets with matched quantitative data. Finally, while we observed correlations between H4K5ac enrichment and gene expression changes, direct causal relationships have not been established. Functional perturbations (e.g., gene knockout or overexpression) are required to validate the regulatory roles of *Itga3* and *Gsk3b*.

Addressing these limitations in future studies will strengthen the mechanistic understanding of epigenetic regulation in NTDs. Our future research will focus on two directions. First, we will validate the biomarker potential of key focal adhesion pathway proteins in larger cohorts and more physiologically relevant disease models. Second, to further verify the function of this epigenetic regulatory axis in neural tube development at both the cellular and animal levels, we will establish cellular perturbation models of genes such as *Itga3* or *Gsk3b*, combined with small-molecule inhibitor interventions and phenotypic analyses (e.g., F-actin staining, scratch assays, Transwell migration assays), thereby providing an experimental basis for the prevention and treatment of related diseases.

## 5. Conclusions

In summary, our findings demonstrate that a maternal hyperglycemic environment disrupts the expression of key focal adhesion pathway proteins, leading to impaired cytoskeletal dynamics and aberrant tissue morphogenesis, thereby contributing to the pathogenesis of NTDs. Furthermore, we reveal that H4K5ac participates in this pathological process by regulating the expression of the core pathway genes *Itga3* and *Gsk3b*. These results provide new insights into the epigenetic mechanisms underlying diabetes-induced NTDs and suggest that targeting the H4K5ac-*Itga3*/*Gsk3b* regulatory axis may represent a potential strategy for preventing or treating hyperglycemia-associated congenital abnormalities.

## Figures and Tables

**Table 1:** Primers for amplification of genes in RT-qPCR.

Genes	Forward Primer	Reverse Primer
*Itga3*	5′-AACTCCGTCCTATCGTCATTGC-3′	5′-TAGCCTGTGCCTGGTTGAG-3′
*Lamc1*	5′-GCAGCCTTCCTGACCGACTACA-3′	5′-GCGTGAGGTTGATGGAGTTGG-3′
*Gsk3b*	5′-AGCCACAGGACAAGGAGAACC-3′	5′-AGCCGAGCGACCTGGATAAC-3′
*Ccnd3*	5′-TGAACTACCTGGATCGCTACCT-3′	5′-CACAGCCTGGTCCGTATAGATG-3′
*β-actin*	5′-GAGAGGGAAATCGTGCGTGACA-3′	5′-AACCGCTCGTTGCCAATAGTGA-3′

**Table 2:** Characteristics of H4K5ac ChIP-seq enrichment in NTDs-related genes.

	Genes	NTDs_group_counts	CON_group_counts	Log2FC	Regulated	Location
up-regulated genes	*Birc5*	48	24	1.600783264	Up	Promoter
	*Itga3*	24	0	7.926007906	Up	Distal Intergenic
	*Hira*	29	0	8.198003262	Up	Intron
	*Cwc22*	35	25	1.087674251	Up	Intron
	*Cox18*	25	0	7.984664291	Up	Distal Intergenic
down-regulated genes	*Casp3*	0	55	−8.513562715	Down	Promoter
	*Cfl1*	0	92	−9.254176449	Down	Promoter
	*Marcksl1*	0	34	−7.822102029	Down	Intron
	*Sfrp2*	0	39	−8.019223854	Down	Promoter
	*Ranbp1*	0	28	−7.543359557	Down	Promoter
	*Dnm1*	0	54	−8.487163606	Down	Promoter
	*Hgs*	0	55	−8.513562715	Down	Promoter
	*Gys1*	0	56	−8.539487429	Down	Promoter
	*Mapk9*	0	34	−7.822102029	Down	Promoter
	*Gsk3b*	0	37	−7.943575561	Down	Intron

Genes with differential H4K5ac binding were identified by thresholds of fold change (FC) > 2 (up-regulated) and FC < 0.5 (down-regulated), combined with the *p*-value < 0.0045.

## Data Availability

The original contributions presented in the study are included in the article, further inquiries can be directed to the corresponding authors.

## Abbreviations

The following abbreviations are used in this manuscript:

H4K5ac	Histone H4 lysine 5 acetylation
NTDs	Neural tube defects
FVB mice	Friend leukemia virus B strain mice
STZ	Streptozotocin
TMT	Tandem mass tag
LC-MS/MS	Liquid chromatography-tandem mass spectrometry
PPI network	Protein–protein interaction network
PGDM	Pregestational diabetes mellitus
ct/ct mice	curly tail homozygous mice
ATRA	All-trans retinoic acid
MALDI-TOF MS	Matrix-assisted laser desorption/ionization time-of-flight mass spectrometry
LC/MS	Liquid chromatography-mass spectrometry
IP-MS	Immunoprecipitation-mass spectrometry
SIRT	Sirtuin
FDR	False discovery rate
ChIP-seq	Chromatin immunoprecipitation followed by sequencing
RT-qPCR	Reverse Transcription-Quantitative Polymerase Chain Reaction
FC	Fold change
DEPs	Differentially expressed proteins
GO annotation	Gene Ontology annotation
ECM	Extracellular matrix
DEGs	Differentially expressed genes
FAK	Focal adhesion kinase
Src	Proto-oncogene tyrosine-protein kinase Src
Gsk3b	Glycogen synthase kinase 3 beta
Itga3	Integrin alpha 3
Mapk9	Mitogen-activated protein kinase 9
MACS	Model-based Analysis of ChIP-Seq
RELN	Reelin
SLMAP3	Sarcolemmal membrane-associated protein 3
Crmp4	Collapsin response mediator protein 4
Hsp70	Heat shock protein 70
